# Supplementary material for: The Clinical Application of Immunohistochemical Expression of Notch4 Protein in Patients with Colon Adenocarcinoma
Source: Int J Mol Sci. 2023 Apr 19;24(8):7502. doi: 10.3390/ijms24087502 (PMC10138794; doi:10.3390/ijms24087502)
Supplement: Supplementary file 1 [file ijms-24-07502-s001.zip › ijms-2283128-SI.pdf]

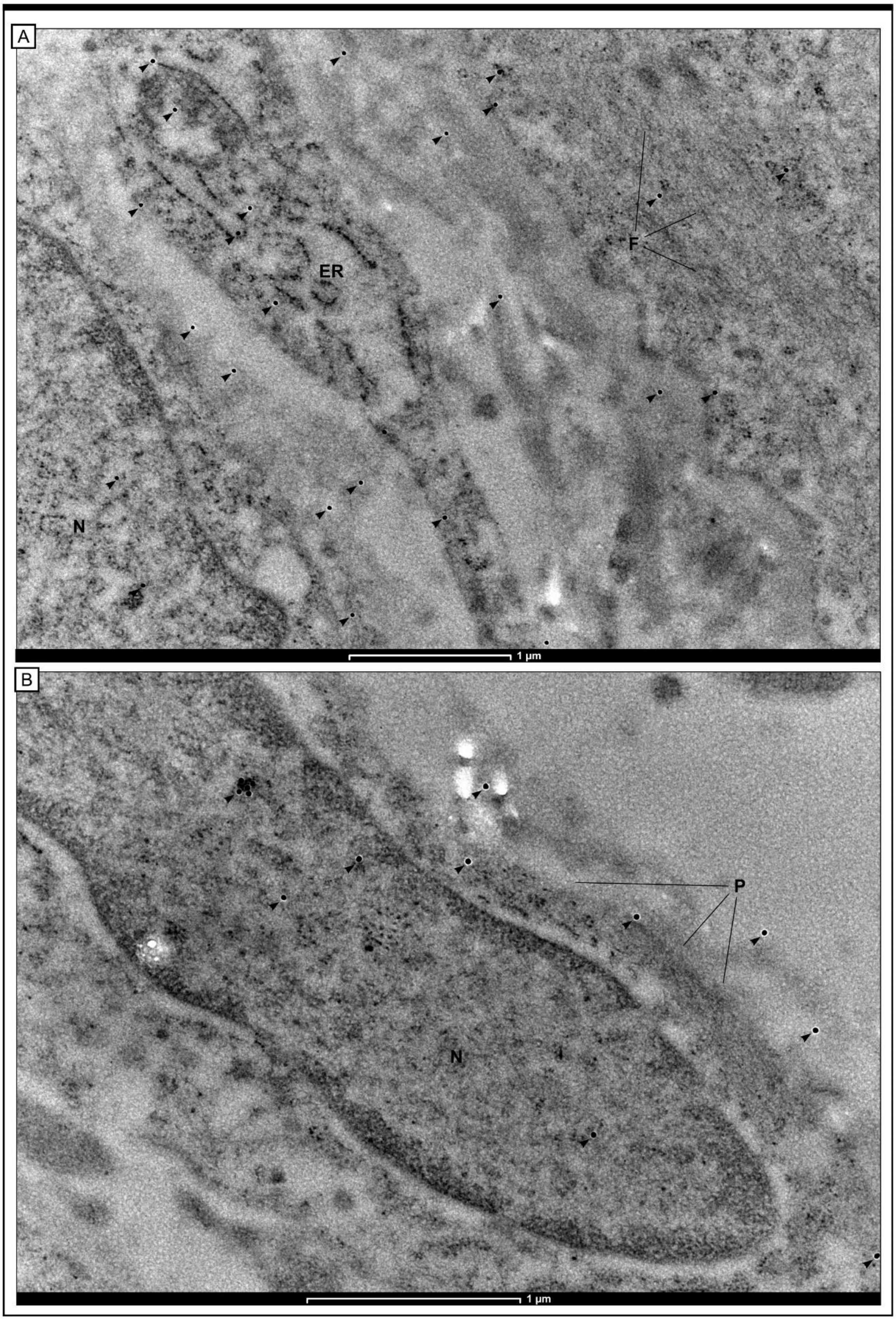

Figure S1: In the fibroblasts of non-pathological colon tissue, the electron-dense black granules indicating the presence of Notch 4 expression (arrowheads) were found in the cell membrane (P), in the nucleus (N) and the endoplasmic reticulum (ER). F- cytoplasmic intermediate filaments. The scale bar is 1 μm (A, B).
